# Genetic Variations in Susceptibility to Traumatic Muscle Injuries and Muscle Pain among Brazilian High-Performance Athletes

**DOI:** 10.3390/ijms25063300

**Published:** 2024-03-14

**Authors:** Inês Soares Marques, Valéria Tavares, Beatriz Vieira Neto, Lucas Rafael Lopes, Rodrigo Araújo Goes, João António Matheus Guimarães, Jamila Alessandra Perini, Rui Medeiros

**Affiliations:** 1 Molecular Oncology and Viral Pathology Group, Research Center of IPO Porto (CI-IPOP)/Pathology and Laboratory Medicine Dep. Clinical Pathology SV/RISE@CI-IPOP (Health Research Network), Portuguese Oncology Institute of Porto (IPO Porto)/Porto Comprehensive Cancer Centre (Porto.CCC), 4200-072 Porto, Portugal; inesmarques1405@gmail.com (I.S.M.); valeria.tavares@ipoporto.min-saude.pt (V.T.); beapedro7@hotmail.com (B.V.N.); 2 Faculty of Sciences of University of Porto (FCUP), 4169-007 Porto, Portugal; 3 Faculty of Medicine of University of Porto (FMUP), 4200-072 Porto, Portugal; 4 ICBAS—Instituto de Ciências Biomédicas Abel Salazar, Universidade do Porto, 4099-002 Porto, Portugal; 5 Research Department, Portuguese League Against Cancer (NRNorte), 4200-172 Porto, Portugal; 6 Pharmaceutical Sciences Research Laboratory (LAPESF), State University of Rio de Janeiro (UERJ), Rio de Janeiro 23070-200, Brazil; lopes.rlucas02@gmail.com (L.R.L.); jamilaperini@yahoo.com.br (J.A.P.); 7 Programa de Pós-Graduação em Saúde Pública e Meio Ambiente, Escola Nacional de Saúde Pública, Fundação Oswaldo Cruz (Fiocruz), Rio de Janeiro 21040-900, Brazil; 8 Research Division, Instituto Nacional de Traumatologia e Ortopedia (INTO), Rio de Janeiro 20940-070, Brazil; rodrigogoes4@yahoo.com.br (R.A.G.); jmatheusguimaraes@gmail.com (J.A.M.G.); 9 University Clinic of Orthopedics and Traumatology, Faculty of Medicine of Lisbon, 1649-028 Lisbon, Portugal; 10 Faculty of Health Sciences, Fernando Pessoa University, 4200-150 Porto, Portugal

**Keywords:** sports, wounds and injuries, pain, sports medicine, preventive medicine, polymorphism, single nucleotide

## Abstract

Traumatic muscle injuries (TMIs) and muscle pain (MP) negatively impact athletes’ performance and quality of life. Both conditions have a complex pathophysiology involving the interplay between genetic and environmental factors. Yet, the existing data are scarce and controversial. To provide more insights, this study aimed to investigate the association of single-nucleotide polymorphisms (SNPs) previously linked to athletic status with TMI and MP after exercise among Brazilian high-performance athletes from different sports modalities (N = 345). The impact of important environmental determinants was also assessed. From the six evaluated SNPs (*ACTN3* rs1815739, *FAAH* rs324420, *PPARGC1A* rs8192678, *ADRB2* rs1042713, *NOS3* rs1799983, and *VDR* rs731236), none was significantly associated with TMI. Regarding MP after exercise, *ACTN3* rs1815739 (CC/CT vs. TT; adjusted odds ratio (aOR) = 1.90; 95% confidence interval (95%Cl), 1.01–3.57) and *FAAH* rs324420 (AA vs. AC/CC; aOR = 2.30; 95%Cl, 1.08–4.91) were independent predictors according to multivariate binomial analyses adjusted for age (≥23 vs. <23 years), sex (male vs. female), and tobacco consumption (yes vs. no). External validation is warranted to assess the predictive value of *ACTN3* rs1815739 and *FAAH* rs324420. This could have implications for prophylactic interventions to improve athletes’ quality of life.

## 1. Introduction

The musculoskeletal system is an intricate framework primarily consisting of the skeletal structure and muscles, while also encompassing ligaments and tendons. It represents the backbone of form, stability, support and movement of the human body [1,2]. However, the function and overall effectiveness of this system can be easily disturbed, mainly by physical injury, causing muscle pain (MP) and potentially impairing the daily activity of individuals [3]. Importantly, MP can manifest as acute (sudden and severe) or chronic (long-lasting) [4,5].

Naturally, the musculoskeletal system has a key role in sports practice [6]. At the highest level of competition (i.e., elite sports), musculoskeletal injuries represent the most common health problem, imposing different levels of impact (both acute and chronic), with some leading to enduring disability [7,8,9]. The prevalence of musculoskeletal lesions in elite athletes is around 80%. Although the specific type and location may vary depending on the sport, muscle injuries are the most frequently observed lesions in this subpopulation, constituting 55% of cases [10]. These lesions comprise strains, sprains, contusions, dislocations, and rupture, with the former and the latter being more prevalent [7]. Consequences of traumatic muscle injuries (TMIs) include the lowering of athletic performance, withdrawal from important competitions, and lifetime disability [9,11]. Moreover, TMI may also affect athletes’ mental health and resilience, and a huge economic burden is associated with diagnosing and treating these lesions [12,13,14,15]. Identifying those at an increased risk for this type of injury and MP after exercise may help tailor prophylactic measures. Thus, a better understanding of the underlying biological mechanisms is required.

Over the last few years, genetic factors, particularly, single-nucleotide polymorphisms (SNPs), have been associated with athletic performance. Their impact on the susceptibility to sports-related lesions is, however, less explored [16,17]. The gene *actinin alpha 3* (*ACTN3*), popularly referred to as the “speed gene,” has been consistently connected to athletic performance through its modulation of sports-related phenotypes, including training adaptation and recovery, as well as the risk of injury [18,19,20]. *Fatty acid amide hydrolase* (*FAAH*) encodes for a protein with the same name, which is associated with inflammation, stress, and pain tolerance [21,22,23,24]. *PPARG coactivator 1 alpha* (*PPARGC1A*) also encodes a protein with the same name, which is thought to regulate muscle fiber composition and training-induced muscle adaptation [25,26,27,28]. Likewise, *adrenoceptor beta 2* (*ADRB2*) is implicated in several functions concerning the central nervous, cardiovascular, endocrine, and pulmonary systems, all affecting athletic performance [26,29,30]. *Nitric oxide synthase 3* (*NOS3*) is a gene with roles in endothelium activity, endurance performance, and athletes’ susceptibility to lesions [26,31,32]. *Vitamin D receptor* (*VDR*) was previously associated with stress fractures among athletes [26,33]. These genes harbor SNPs that, combined with environmental cues, could be involved in both TMI and MP after exercise in the setting of athletic performance. To offer a deeper understanding, a case-control study was designed to evaluate the association between relevant SNPs and the susceptibility to TMI and MP after exercise among high-performance athletes, considering environmental influences.

## 2. Results

### 2.1. Characterization of Study Population

**Figure 1:**
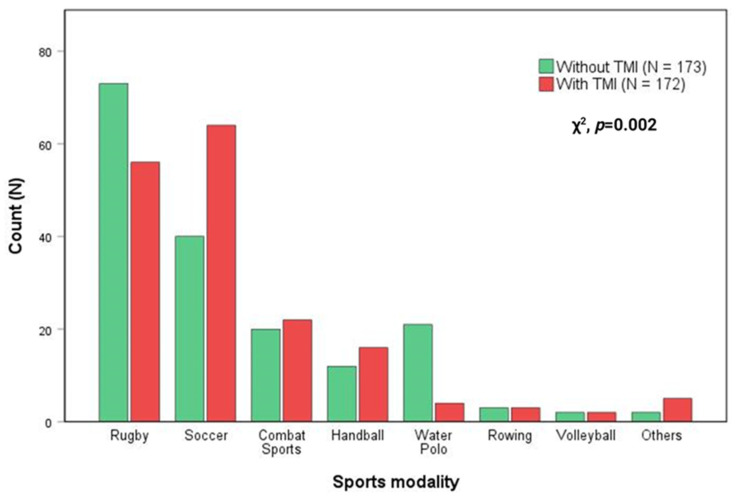
Status of traumatic muscle injury (TMI) across different sports modalities (N = 345). TMI occurrence among high-performance athletes varies significantly depending on the sports modality (Chi-squared test (χ^2^), *p* = 0.002).

This study enrolled 345 Brazilian high-performance athletes with (N = 172) and without (N = 173) TMI. The cohort characterization is provided in Table 1. The enrolled athletes participated in various sports disciplines: 129 from rugby, 104 from soccer, 42 from combat sports, 28 from handball, 25 from water polo, 6 from rowing, 4 from volleyball, and 7 from other sports modalities (Figure 1).

### 2.2. Distribution of SNP Genotypes

The distribution of the evaluated polymorphisms is represented in Table 2. Although the Brazilian population is thought to have considerable ancestral heterogeneity, the genomic ancestry of individuals from different regions in Brazil was found to be more homogeneous than first assumed [34]. The genotype frequencies of the evaluated SNPs in this cohort were compared with the reported distribution by other studies with the Brazilian population. For each study, the overall population was considered, i.e., all individuals with and without the trait of interest. The six SNPs were in Hardy–Weinberg equilibrium (χ^2^, *p* > 0.05), indicating no significant difference in the frequency distribution of the SNPs’ genotypes compared to the frequency described in the literature involving Brazilians from different country regions [35,36,37,38,39,40].

### 2.3. Associations between the SNPs and the Athletes’ Characteristics

No significant statistical differences in the distribution of the SNPs’ genotypes according to the athletes’ characteristics were observed, except for *adrenoceptor beta 2* (*ADRB2*) rs1042713 and *vitamin D receptor gene* (*VDR*) rs731236. Specifically, a significant association between *ADRB2* rs1042713 and the athletes’ age (≥23 vs. <23 years) was detected (GG vs. GA vs. AA, *p* = 0.027; GG vs. GA/AA, *p* = 0.010). Namely, the GG genotype was predominant among those aged 23 years or older, while the A allele genotypes were more common within the younger athletes (61.4% and 53.9%, respectively). The distribution of *VDR* rs731236 genotypes significantly varied according to tobacco consumption (AA vs. GA/GG, *p* = 0.040) and the athletes’ sex (AA/GA vs. GG, *p* = 0.044). Specifically, the G allele genotypes were more predominant among the smokers than the non-smokers (84.2% and 57.5%, respectively). Furthermore, although the GG genotype had almost the same frequency between males and females (52.8% and 47.2%, respectively), the A allele genotypes were mostly present among male athletes (70.8%).

### 2.4. Associations of the SNPs with TMI and MP after Exercise

In univariable binomial regression analyses, no significant association between the evaluated SNPs and TMI occurrence was observed (*p* > 0.05). In terms of the athletes’ characteristics, age (≥23 vs. <23 years; odds ratio (OR) = 2.42; 95% confidence interval (95%Cl), 1.57–3.73; *p* < 0.001) and training experience (≥9 vs. <9 years; OR = 2.41; 95%Cl, 1.56–3.71; *p* < 0.001) were linked to TMI development. In multivariate binomial regression analyses considering these athletes’ characteristics, no association between the SNPs and TMI was detected (*p* > 0.05).

Regarding MP after exercise, a significant association was observed for *FAAH* rs324420. Individuals carrying the AA genotype (A is the minor allele) were two times more prone to MP than those with the C allele (AA vs. AC/CC; OR = 2.20; 95%Cl, 1.04–4.65; *p* = 0.039). Additionally, a marginal association was detected for *ACTN3* rs1815739. Specifically, athletes with the C allele tend to be more susceptible to MP after exercise than TT genotype carriers (CC/CT vs. TT; OR = 1.86; 95%Cl, 1.00–3.48; *p* = 0.051). Considering the remaining evaluated SNPs, namely, *PPARGC1A* rs8192678, *ADRB2* rs1042713, *NOS3* rs1799983, and *VDR* rs731236, no significant association was identified (*p* > 0.05). As for the athletes’ characteristics, tobacco consumption was the only predictor of MP after exercise (yes vs. no; OR = 2.65; 95%Cl, 1.02–6.91; *p* = 0.046). In multivariable binomial regression analyses adjusted for the athletes’ age, sex, and tobacco consumption, *FAAH* rs324420 and *ACTN3* rs1815739 were confirmed to be independent predictors of MP after exercise (Table 3).

## 3. Discussion

Beyond environmental factors, particularly, training conditions and nutrition, athletic performance is determined by the individual’s genetic architecture [18]. Indeed, the influence of inherited traits in sports performance has been an attractive research field during the last few decades, with recent studies exploring the genetic contribution underlying sports-related injuries [16]. Given the growing interest in this topic, the present study aimed to evaluate the association between relevant SNPs and the susceptibility to TMI and MP after exercise among high-performance athletes, not dismissing environmental influences.

Starting with TMI, none of the six evaluated SNPs were significantly associated with this condition (*p* > 0.05). In terms of the athletes’ characteristics, as expected, age and training experience were linked to TMI development. Specifically, older athletes (≥23 vs. <23 years; OR = 2.42; 95%Cl, 1.57–3.73) and those with more training experience (≥9 vs. <9 years; OR = 2.41; 95%Cl, 1.56–3.71) were two times more prone to lesions. Regarding MP after exercise, tobacco consumption was the only predictor. Namely, smokers were almost three times more susceptive to MP than nonsmokers (yes vs. no; OR = 2.65; 95%Cl, 1.02–6.91; *p* = 0.046). Indeed, the use of tobacco has been demonstrated to have negative repercussions on the musculoskeletal system [41]. Due to direct toxic effects, tobacco can diminish bone mineral content, reduce muscle mass strength, and increase the number of fractures and the risk of MP [41,42]. Thus, the study results are in line with the current evidence. Furthermore, the SNPs *ACTN3* rs1815739 (R577X) and *FAAH* rs324420 (C385A) were found to be independent predictors of MP after exercise according to the multivariate analyses adjusted for the athletes’ age, sex, and tobacco use.

ACTN3 is a sarcomeric protein that anchors actin filaments to the Z-line in fast-twitch type 2 muscle fibers. By generating force at high speed, these fibers promote short and explosive periods of physical activity [43,44]. In addition to muscle performance, α-actinin-3 is also implicated in several metabolic and signaling pathways due to its interaction with multiple macromolecules [45]. The R577X SNP is defined by the substitution of a cytosine (C) to a thymine (T) at nucleotide position 1747. This change (C > T) results in the conversion of an arginine (R allele) to a premature stop codon (X allele) at residue 577, which translates into the expression of a truncated and non-functional protein [26,46,47]. Importantly, TT and CC genotypes had a frequency consistent with that reported in the literature for Brazilian individuals [35]. Carriers of the 577XX genotype (or TT genotype) are known to be deficient in α-actinin-3 and consequently have a lower fast-twitch fiber percentage, meaning lower fast type 2 muscle function [46]. Interestingly, around 18% of healthy white individuals and about 16% of people worldwide have this genotype, and it is more common in the general population than in sprint and power athletes [43,44,48]. As for those with the 577R allele (C allele), the varied expression levels of α-actinin-3 at certain conditions can affect muscle performance differently [48]. Indeed, previous studies suggested that *ACTN3* genotypes have the potential to change the functioning of the skeletal muscle through metabolic, structural, or signaling-dependent mechanisms [18,44,49,50]. As expected, these modifications have implications for the overall performance of athletes, as they also affect their risk of TMI and MP [44,51,52]. Power athletes exhibit a higher prevalence of the C allele (associated with α-actinin-3 expression), suggesting that α-actinin-3 is imperative in swiftly optimizing muscle function. In opposition, the TT genotype (related to α-actinin-3 deficiency) is usually found among endurance athletes, indicating that the protein absence might benefit long-distance performance [47]. Taken together, the SNP seems to have a sports modality-related effect. Although the link to sports-related lesions has been explored, the association between *ACTN3* SNP and MP remains less understood. The scarce data indicate a protective role of the R577X C allele [51,53]. In the present study, however, athletes carrying the SNP C allele were two times more prone to MP than their counterparts, suggesting a detrimental role. This conflicting result could be explained by the sports modality-related effect of the genetic variant. In this study, the insufficient statistical power prevented stratified analysis based on the sports type, which would also be important given the different distribution of lesions according to sports group [10]. 

By regulating the neuronal excitability in the amygdala, a brain area that regulates anxiety, FAAH is thought to modulate anxiety-related behavior [54,55]. Also, via the same system, inhibition of FAAH suppresses pathological pain [28,56]. Regarding rs324420, this missense SNP is defined by the substitution of a cytosine (C) to an adenine (A), which results in a higher sensitivity of the encoded protein to proteolytic degradation. In fact, the 385A allele (A allele; minor allele) has been associated with diminished FAAH levels and, thus, higher tolerance to pain [54,55,56]. Interestingly, the same allele was previously linked to better athletic achievements in studies conducted by our research group with rink-hockey and volleyball players [22,23]. In the present study, however, athletes carrying the rs324420 AA genotype were two times more prone to MP after exercise than their counterparts. Given the scarce published data, the mechanisms underlying this finding need to be further dissected. Collectively, *FAAH* rs324420 may be a potential tool to assess the athletes’ susceptibility to MP and their performance. Whether its implications depend on sports type is a matter of discussion.

Regarding *PPARGC1A* rs8192678, *ADRB2* rs1042713, *NOS3* rs1799983, and *VDR* rs731236, no significant association with TMI or MP was detected in univariable or multivariable analyses (*p* > 0.05). Worth mentioning is that the distribution of *ADRB2* rs1042713 genotypes was different depending on the age group, which was one of the athlete characteristics that was significantly associated with TMI susceptibility. Likewise, *VDR* rs731236 genotypes were distributed differently depending on tobacco use. Indeed, vitamin D deficiency has been linked to tobacco consumption [57,58]. Whether *VDR* rs731236 also contributes to this deficiency among the exposed individuals or, inversely, whether the SNP somehow influences tobacco consumption requires further investigation. The *VDR* rs731236 genotypes were also distributed differently according to the athletes’ sex, which was previously observed in a study conducted by our research group [22]. Taken together and considering the roles of these SNPs, future studies with larger cohort sizes should be conducted to reevaluate the impact of these genetic variants on TMI and MP susceptibility among high-performance athletes.

In terms of the study limitations, the small cohort size and the inability to conduct stratified analysis considering the different sports modalities might have prevented the detection of additional associations. The latter would be important given that each sport has its own requirements and a unique profile of injuries. 

## 4. Materials and Methods

### 4.1. Athlete Recruitment

For this study, 345 Brazilian athletes with (cases; N = 172) and without (controls; N = 173) TMI were enrolled. The inclusion criteria incorporated athletes aged between 18 and 45 years and whose history of TMI was attributed to sports practice. The recruitment was conducted from March 2018 to December 2019 and involved different sports modalities. All TMI diagnoses were blindly confirmed by two specialized orthopedists. A questionnaire regarding demographic, clinical, sports, and training characteristics was requested of each participant and further checked in the presence of an expert researcher. The questionnaire characteristics were previously defined elsewhere [10].

### 4.2. SNP Selection

More than 80 genetic determinants have been associated with endurance, power, and/or sports injuries among athletes across different sports modalities [16]. Focusing on the SNPs with roles in muscle function and/or performance, pain, inflammation, and metabolic pathways, the selection of the most suitable SNPs to be evaluated was conducted considering their minor allele frequency (MAF) reported in previous studies with the Brazilian population (an MAF of at least 15% was considered), their functional consequence, and the availability of predesigned TaqMan^TM^ SNP Genotyping Assays (Applied Biosystems). By applying these criteria, six SNPs were selected, namely, rs1815739 in *ACTN3*, rs324420 in *FAAH*, rs8192678 in *PPARGC1A*, rs1042713 in *ADRB2*, rs1799983 in *NOS3*, and rs731236 in *VDR*.

### 4.3. Sample Collection, Genomic DNA Extraction, and SNP Genotyping 

Genomic deoxyribonucleic acid (DNA) of each athlete was extracted from saliva samples as previously described [59]. DNA concentration and purity were assessed using a Nanodrop^®^ spectrophotometer (Thermo Scientific^®^, Wilmington, DE, USA).

SNP genotyping was performed in a StepOne Plus Real-Time Polymerase Chain Reaction (real-time PCR) system (Applied Biosystems^®^, Foster City, CA, USA). The TaqMan^®^ Allelic Discrimination methodology was employed with the use of predesigned TaqMan^TM^ SNP Genotyping Assays (Applied Biosystems^®^, Foster City, CA, USA). Each PCR reaction was conducted using 2.5 µL of TaqPath^TM^ ProAmp^TM^ Master Mix (1×) (Applied Biosystems^®^, Foster City, CA, USA); 2.375 µL of sterile water; 0.125 µL of TaqMan^TM^ SNP Genotyping assay (*ACTN3* rs1815739 (C____590093_1_), *FAAH* rs324420 (C___1897306_10), *PPARGC1A* rs8192678 (C___1643192_20), *ADRB2* rs1042713 (C___2084764_20), *NOS3* rs1799983 (C___3219460_20), and *VDR* rs731236 (C___2404008_10); and 1.0 µL of genomic DNA, in a total volume of 6 µL. The thermal cycling conditions for DNA amplification were described elsewhere [60]. Analysis of DNA amplification was employed by StepOne Software (version 2.3 Applied Biosystems). In each PCR run, negative controls (without DNA) were included to assess false positives. A double sampling of 20% of randomly selected DNA samples was conducted and complete concordance was confirmed. The evaluation of genotyping results was made by three researchers engaged in the study but blinded to the demographic, clinical, sports, and training characteristics of the athletes.

### 4.4. Statistical Analyses

Statistical analyses were performed using IBM^®^ SPSS^®^ Statistics software package version 28.0.0.0 (IBM Corp., Armonk, NY, USA, Released 2021). The distribution of the SNPs’ genotypes in the Brazilian population was assessed and the Hardy–Weinberg equilibrium (HWE) was tested employing the Chi-square test (χ^2^). The Kolmogorov–Smirnov test was used to evaluate the distribution of variables. Associations between the SNPs (considering the additive, recessive, and dominant genetic models) and the athletes’ demographic, clinical, sports, and training factors were assessed using the χ^2^ test for categorical variables, whereas for continuous ones, either the Mann–Whitney U test or the Student’s *t*-test was employed (for not-normal and normally distributed variables, respectively). The list of factors included the athletes’ age (years), sex (male vs. female), body mass index (BMI; kg/m^2^), tobacco consumption (yes vs. no), alcohol consumption (yes vs. no), age at sports practice initiation (years), training experience (years), training frequency (hours/week), and the level of sports competition (school/university vs. professional). The athletes’ age (≥23 vs. <23 years), BMI (≥25 vs. <25 kg/m^2^), age at sports practice initiation (≥14 vs. <14 years), training experience (≥9 vs. <9 years), and training frequency (≥12 vs. <12 h/week) were also assessed as nominal variables. These variables’ categories were defined based on the median value, as they were not normally distributed (Kolmogorov–Smirnov test, *p* < 0.05).

Univariable binomial regression analyses were performed to identify SNPs associated with the occurrence of TMI and MP after exercise. In these analyses, a *p*-value higher than 0.05 but lower than 0.06 was considered marginally significant. Focusing on the relevant SNPs, multivariable binomial regression analyses were conducted, adjusting for the athletes’ age, sex, and other factors significantly linked to the respective trait (TMI and MP after exercise) in the univariable binomial regression analyses.

## 5. Conclusions

In this case-control study, six SNPs previously linked to athletic performance and with roles in muscle function and/or performance, pain, inflammation, and metabolic pathways were evaluated regarding their impact on the susceptibility for TMI and MP after exercise among Brazilian high-performance athletes across various sports modalities. Although no significant association between the SNPs and TMI was observed, *ACTN3* rs1815739 and *FAAH* rs324420 were found to be independent predictors of MP after exercise. While additional studies are required to replicate and validate the study findings in larger populations that are not primarily based in Brazil, these polymorphisms might constitute useful and valuable biomarkers for personalized training programs to not only optimize performance but also improve the quality of life of athletes. Furthermore, studies focusing on the specific sports modalities are encouraged given that each sport possesses different requirements that might influence the relevance of these SNPs.

## Figures and Tables

**Table 1:** Athletes’ characteristics according to the occurrence of TMI.

Characteristics	Athletes			χ^2^ *p*-Value
	With TMI (N = 172) N (%)	Without TMI (N = 173) N (%)	Total (N = 345) N (%)	
Age (years) *	25.5 ± 5.9	22.4 ± 4.5	23.9 ± 5.4	**<0.001**
**Sex**				
Female	49 (28.5)	59 (34.1)	108 (31.3)	0.261
Male	123 (71.5)	114 (65.9)	237 (68.7)	
BMI (kg/m^2^) *	24.8 ± 3.5	24.4 ± 3.4	24.6 ± 3.4	0.294
Tobacco consumption **	11 (6.4)	8 (4.6)	19 (5.5)	0.471
Alcohol consumption **	98 (57.0)	97 (56.1)	195 (56.5)	0.865
**Age at sports practice initiation (years)**	13.4 ± 6.5	14.3 ± 6.0	13.8 ± 6.2	0.190
Training experience (years)*	11.2 ± 6.6	8.1 ± 5.7	9.7 ± 6.4	**<0.001**
Training frequency (hours/week) *	13.5 ± 9.0	12.4 ± 6.6	13.0 ± 7.9	0.214
**Level of sports competition**				
School/university	19 (11.0)	25 (14.5)	44 (12.8)	0.343
Professional	153 (89.0)	148 (85.5)	301 (87.2)	
**MP after exercise**	87 (50.6)	53 (30.6)	140 (40.6)	**<0.001**

* Data presented as mean ± standard deviation. ** Defined as both past and active consumption. Bold values represent significant results (*p* < 0.05) between TMI groups obtained through the Chi-squared test (χ^2^ *p*-value) or Fisher’s exact test. Abbreviations: BMI, body mass index; TMI, traumatic muscle injury; MP, muscle pain.

**Table 2:** Genotype frequencies of the evaluated SNPs in the study population (N = 345) and the frequencies reported by previous studies with the Brazilian population.

Polymorphism Genotype	Study Population ^a^			Previous Studies with Brazilian Population			χ^2^ *p*-Value
	Genotype Frequency	Failed Genotyping	MAF (MA)	Genotype Frequency	MAF (MA)	Reference ^b^	
***ACTN3* rs1815739**							
TT	56 (16.2)	1 (0.3)	40.9 (T)	120 (19.9)	41.9 (T)	[35]	0.313
CT	165 (47.8)			265 (44.0)			
CC	123 (35.7)			217 (36.0)			
***FAAH* rs324420**							
AA	31 (9.0)	1 (0.3)	26.9 (A)	14 (7.0)	25.0 (A)	[36]	0.708
AC	123 (35.7)			72 (36.0)			
CC	190 (55.1)			114 (57.0)			
***PPARGC1A* rs8192678**							
TT	31 (9.0)	2 (0.6)	27.1 (T)	15 (6.0)	25.1 (T)	[37]	0.409
CT	124 (35.9)			94 (38.1)			
CC	188 (54.5)			138 (55.9)			
***ADRB2* rs1042713**							
AA	69 (20.0)	3 (0.9)	43.4 (A)	8 (11.4)	37.9 (A)	[38]	0.226
AG	159 (46.1)			37 (52.9)			
GG	114 (33.0)			25 (35.7)			
***NOS3* rs1799983**							
TT	28 (8.1)	1 (0.3)	26.9 (T)	13 (6.1)	23.0 (T)	[39]	0.414
GT	129 (37.4)			73 (34.3)			
GG	187 (54.2)			127 (59.6)			
***VDR* rs731236**							
GG	36 (10.4)	1 (0.3)	34.7 (G)	12 (8.1)	29.7 (G)	[40]	0.271
AG	167 (48.4)			64 (43.2)			
AA	141 (40.9)			72 (48.6)			

^a^ The study population was recruited in the State of Rio de Janeiro (southeastern region). ^b^ The study populations in previous studies on *ACTN3* rs1815739, *FAAH* rs324420, *PPARGC1A* rs8192678, *ADRB2* rs1042713, *NOS3* rs1799983, and *VDR* rs731236 were enrolled in various regions of Brazil [35] and the southeastern [36,37,38], northeastern [39], and northern regions [40] of Brazil. Abbreviations: MA, minor allele; MAF, minor allele frequency; SNPs, single-nucleotide polymorphisms.

**Table 3:** Multivariable binomial regression analyses on the susceptibility to muscle pain (MP) after exercise (N = 344) among athletes according to *ACTN3* rs1815739 and *FAAH* rs324420.

**Variable**	**Adjusted OR**	**95% CI**	***p*** **-Value**
Age (≥23 vs. <23 years ^1^) *	0.93	(0.60–1.45)	0.756
Sex (male vs. female ^1^)	0.94	(0.58–1.50)	0.779
Tobacco consumption (yes vs. no ^1^)	2.74	**(1.04–7.27)**	**0.042**
*ACTN3* rs1815739 (CC/CT vs. TT ^1^)	1.90	**(1.01–3.57)**	**0.047**
**Variable**	**Adjusted OR**	**95% CI**	***p*** **-Value**
Age (≥23 vs. <23 years ^1^) *	0.95	(0.61–1.47)	0.804
Sex (male vs. female ^1^)	0.92	(0.57–1.48)	0.729
Tobacco consumption (yes vs. no ^1^)	2.82	**(1.07–7.45)**	**0.037**
*FAAH* rs324420 (AA vs. AC/CC ^1^)	2.30	**(1.08–4.91)**	**0.031**

^1^ Reference group. * Cut-off defined based on variable median values. Bold values represent significant results (*p* < 0.05) obtained through the binomial regression analyses. Abbreviations: CI, confidence interval; OR, odds ratio.

## Data Availability

Data will be made available on reasonable request.

## References

[1] Ni Y., Gao Y., Yao J. (2022). Introduction to musculoskeletal system. Biomechanical Modelling and Simulation on Musculoskeletal System.

[2] Knudson D. (2021). Mechanics of the musculoskeletal system. Fundamentals of Biomechanics.

[3] Søgaard K., Sjøgaard G. (2017). Physical activity as cause and cure of muscular pain: Evidence of underlying mechanisms. Exerc. Sport Sci. Rev..

[4] Katz W. (2002). Musculoskeletal pain and its socioeconomic implications. Clin. Rheumatol..

[5] Arendt-Nielsen L., Fernández-de-Las-Peñas C., Graven-Nielsen T. (2011). Basic aspects of musculoskeletal pain: From acute to chronic pain. J. Man. Manip. Ther..

[6] Bliss S., Zink C., Van Dyke J. (2018). Musculoskeletal structure and physiology. Canine Sports Medicine and Rehabilitation.

[7] Greising S.M., Corona B.T., Call J.A. (2020). Musculoskeletal regeneration, rehabilitation, and plasticity following traumatic injury. Int. J. Sports Med..

[8] Balogh Z.J., Reumann M.K., Gruen R.L., Mayer-Kuckuk P., Schuetz M.A., Harris I.A., Gabbe B.J., Bhandari M. (2012). Advances and future directions for management of trauma patients with musculoskeletal injuries. Lancet.

[9] Gimigliano F., Resmini G., Moretti A., Aulicino M., Gargiulo F., Gimigliano A., Liguori S., Paoletta M., Iolascon G. (2021). Epidemiology of musculoskeletal injuries in adult athletes: A scoping review. Medicina.

[10] Goes R.A., Lopes L.R., Cossich V.R.A., de Miranda V.A.R., Coelho O.N., do Carmo Bastos R., Domenis L.A.M., Guimarães J.A.M., Grangeiro-Neto J.A., Perini J.A. (2020). Musculoskeletal injuries in athletes from five modalities: A cross-sectional study. BMC Musculoskelet. Disord..

[11] Teyhen D.S., Shaffer S.W., Goffar S.L., Kiesel K., Butler R.J., Rhon D.I., Plisky P.J. (2020). Identification of risk factors prospectively associated with musculoskeletal injury in a warrior athlete population. Sports Health.

[12] Lisman P.J., de la Motte S.J., Gribbin T.C., Jaffin D.P., Murphy K., Deuster P.A. (2017). A Systematic Review of the Association between Physical Fitness and Musculoskeletal Injury Risk: Part 1-Cardiorespiratory Endurance. J. Strength Cond. Res..

[13] Yard E.E., Schroeder M.J., Fields S.K., Collins C.L., Comstock R.D. (2008). The epidemiology of United States high school soccer injuries 2005–2007. Am. J. Sports Med..

[14] Bromley S.J., Drew M.K., Talpey S., McIntosh A.S., Finch C.F. (2018). A systematic review of prospective epidemiological research into injury and illness in Olympic combat sport. Br. J. Sports Med..

[15] Toohey L.A., Drew M.K., Finch C.F., Cook J.L., Fortington L.V. (2019). A 2-Year Prospective Study of Injury Epidemiology in Elite Australian Rugby Sevens: Exploration of Incidence Rates, Severity, Injury Type, and Subsequent Injury in Men and Women. Am. J. Sports Med..

[16] Silva H.-H., Silva M., Cerqueira F., Tavares V., Medeiros R. (2021). Genomic profile in association with sport-type, sex, ethnicity, psychological traits and sport injuries of elite athletes. J. Sports Med. Phys. Fit..

[17] Cornett E.M., Carroll Turpin M.A., Pinner A., Thakur P., Sekaran T.S.G., Siddaiah H., Rivas J., Yates A., Huang G.J., Senthil A. (2020). Pharmacogenomics of pain management: The impact of specific biological polymorphisms on drugs and metabolism. Curr. Oncol. Rep..

[18] Dogan M., Aslan B.T., Ulucan K. (2022). Comparison of potential biomarker, ACTN3 rs1815739 polymorphism, for athletic performance of Turkish athletes. Cell. Mol. Biol..

[19] Digre A., Lindskog C. (2021). The human protein atlas—Spatial localization of the human proteome in health and disease. Protein Sci..

[20] Pickering C., Kiely J. (2017). ACTN3: More than just a gene for speed. Front. Physiol..

[21] Dongdem J.T., Helegbe G.K., Opare-Asamoah K., Wezena C.A., Ocloo A. (2022). Assessment of NSAIDs as potential inhibitors of the fatty acid amide hydrolase I (FAAH-1) using three different primary fatty acid amide substrates in vitro. BMC Pharmacol. Toxicol..

[22] Silva H.-H., Tavares V., Silva M.-R.G., Neto B.V., Cerqueira F., Medeiros R. (2022). FAAH rs324420 Polymorphism Is Associated with Performance in Elite Rink-Hockey Players. Biology.

[23] Silva H.-H., Tavares V., Silva M.-R.G., Neto B.V., Cerqueira F., Medeiros R. (2023). Association of FAAH rs324420 (C385A) Polymorphism with High-Level Performance in Volleyball Players. Genes.

[24] Silva H.-H., Tavares V., Neto B.V., Cerqueira F., Medeiros R., Silva M.-R.G. (2023). FAAH rs324420 Polymorphism: Biological Pathways, Impact on Elite Athletic Performance and Insights for Sport Medicine. Genes.

[25] Lin J., Handschin C., Spiegelman B.M. (2005). Metabolic control through the PGC-1 family of transcription coactivators. Cell Metab..

[26] Howe K.L., Achuthan P., Allen J., Allen J., Alvarez-Jarreta J., Amode M.R., Armean I.M., Azov A.G., Bennett R., Bhai J. (2021). Ensembl 2021. Nucleic Acids Res..

[27] Tharabenjasin P., Pabalan N., Jarjanazi H. (2019). Association of PPARGC1A Gly428Ser (rs8192678) polymorphism with potential for athletic ability and sports performance: A meta-analysis. PLoS ONE.

[28] Kim H., Mittal D., Iadarola M., Dionne R. (2006). Genetic predictors for acute experimental cold and heat pain sensitivity in humans. J. Med. Genet..

[29] Tsianos G.I., Evangelou E., Boot A., Carola Zillikens M., van Meurs J.B., Uitterlinden A.G., Ioannidis J.P. (2010). Associations of polymorphisms of eight muscle-or metabolism-related genes with performance in Mount Olympus marathon runners. J. Appl. Physiol..

[30] Akhmetov I., Popov D., Mozhaĭskaia I., Missina S., Astratenkova I., Vinogradova O., Rogozkin V. (2007). Association of regulatory genes polymorphisms with aerobic and anaerobic performance of athletes. Ross. Fiziol. Zhurnal Im. IM Sechenova.

[31] Sessa F., Chetta M., Petito A., Franzetti M., Bafunno V., Pisanelli D., Sarno M., Iuso S., Margaglione M. (2011). Gene polymorphisms and sport attitude in Italian athletes. Genet. Test. Mol. Biomark..

[32] Kumagai H., Miller B., Kim S.-J., Leelaprachakul N., Kikuchi N., Yen K., Cohen P. (2023). Novel Insights into Mitochondrial DNA: Mitochondrial Microproteins and mtDNA Variants Modulate Athletic Performance and Age-Related Diseases. Genes.

[33] Varley I., Hughes D.C., Greeves J.P., Stellingwerff T., Ranson C., Fraser W.D., Sale C. (2018). The association of novel polymorphisms with stress fracture injury in Elite Athletes: Further insights from the SFEA cohort. J. Sci. Med. Sport.

[34] Pena S.D., Di Pietro G., Fuchshuber-Moraes M., Genro J.P., Hutz M.H., Kehdy F.d.S.G., Kohlrausch F., Magno L.A.V., Montenegro R.C., Moraes M.O. (2011). The genomic ancestry of individuals from different geographical regions of Brazil is more uniform than expected. PLoS ONE.

[35] Cunha A., Nelson-Filho P., Marañón-Vásquez G.A., de Carvalho Ramos A.G., Dantas B., Sebastiani A.M., Silvério F., Omori M.A., Rodrigues A.S., Teixeira E.C. (2019). Genetic variants in ACTN3 and MYO1H are associated with sagittal and vertical craniofacial skeletal patterns. Arch. Oral Biol..

[36] Martins C.J.d.M., Genelhu V., Pimentel M.M.G., Celoria B.M.J., Mangia R.F., Aveta T., Silvestri C., Di Marzo V., Francischetti E.A. (2015). Circulating endocannabinoids and the polymorphism 385C> A in fatty acid amide hydrolase (FAAH) gene may identify the obesity phenotype related to cardiometabolic risk: A study conducted in a Brazilian population of complex interethnic admixture. PLoS ONE.

[37] de Queiroz E.M., Cândido A.P.C., Castro I.d.M., Bastos A.Q.A., Machado-Coelho G., Freitas R.N.d. (2015). IGF2, LEPR, POMC, PPARG, and PPARGC1 gene variants are associated with obesity-related risk phenotypes in Brazilian children and adolescents. Braz. J. Med. Biol. Res..

[38] dos Santos K., Rosado E.L., da Fonseca A.C.P., Belfort G.P., da Silva L.B.G., Ribeiro-Alves M., Zembrzuski V.M., Martínez J.A., Saunders C. (2022). FTO and ADRB2 Genetic Polymorphisms Are Risk Factors for Earlier Excessive Gestational Weight Gain in Pregnant Women with Pregestational Diabetes Mellitus: Results of a Randomized Nutrigenetic Trial. Nutrients.

[39] da Silva T.M., Rocha A.V., Lacchini R., Marques C.R., Silva E.S., Tanus-Santos J.E., Rios-Santos F. (2012). Association of polymorphisms of endothelial nitric oxide synthase (eNOS) gene with the risk of primary open angle glaucoma in a Brazilian population. Gene.

[40] Ferraz R.S., Silva C.S., Cavalcante G.C., de Queiroz N.N., Felício K.M., Felício J.S., Ribeiro-dos-Santos Â. (2022). Variants in the VDR Gene May Influence 25 (OH) D Levels in Type 1 Diabetes Mellitus in a Brazilian Population. Nutrients.

[41] Abate M., Vanni D., Pantalone A., Salini V. (2013). Cigarette smoking and musculoskeletal disorders. Muscles Ligaments Tendons J..

[42] Palmer K.T., Syddall H., Cooper C., Coggon D. (2003). Smoking and musculoskeletal disorders: Findings from a British national survey. Ann. Rheum. Dis..

[43] Murphy A.C., Young P.W. (2015). The actinin family of actin cross-linking proteins–a genetic perspective. Cell Biosci..

[44] Yang N., MacArthur D.G., Gulbin J.P., Hahn A.G., Beggs A.H., Easteal S., North K. (2003). ACTN3 genotype is associated with human elite athletic performance. Am. J. Hum. Genet..

[45] Lee F.X., Houweling P.J., North K.N., Quinlan K.G. (2016). How does α-actinin-3 deficiency alter muscle function? Mechanistic insights into ACTN3, the ‘gene for speed’. Biochim. Et Biophys. Acta (BBA)-Mol. Cell Res..

[46] Vincent B., De Bock K., Ramaekers M., Van den Eede E., Van Leemputte M., Hespel P., Thomis M.A. (2007). ACTN3 (R577X) genotype is associated with fiber type distribution. Physiol. Genom..

[47] MacArthur D.G., North K.N. (2004). A gene for speed? The evolution and function of α-actinin-3. Bioessays.

[48] Harada N., Gotoda Y., Hatakeyama A., Nakagawa T., Miyatake Y., Kuroda M., Masumoto S., Tsutsumi R., Nakaya Y., Sakaue H. (2020). Differential regulation of Actn2 and Actn3 expression during unfolded protein response in C2C12 myotubes. J. Muscle Res. Cell Motil..

[49] Del Coso J., Rodas G., Buil M.Á., Sánchez-Sánchez J., López P., González-Ródenas J., Gasulla-Anglés P., López-Samanes Á., Hernández-Sánchez S., Iztueta A. (2022). Association of the ACTN3 rs1815739 Polymorphism with Physical Performance and Injury Incidence in Professional Women Football Players. Genes.

[50] Demirci B., Bulgay C., Ceylan H.İ., Öztürk M.E., Öztürk D., Kazan H.H., Ergun M.A., Cerit M., Semenova E.A., Larin A.K. (2023). Association of ACTN3 R577X Polymorphism with Elite Basketball Player Status and Training Responses. Genes.

[51] Del Coso J., Hiam D., Houweling P., Pérez L.M., Eynon N., Lucía A. (2019). More than a ‘speed gene’: ACTN3 R577X genotype, trainability, muscle damage, and the risk for injuries. Eur. J. Appl. Physiol..

[52] Massidda M., Voisin S., Culigioni C., Piras F., Cugia P., Yan X., Eynon N., Calò C.M. (2019). ACTN3 R577X polymorphism is associated with the incidence and severity of injuries in professional football players. Clin. J. Sport Med..

[53] Del Coso J., Valero M., Salinero J.J., Lara B., Díaz G., Gallo-Salazar C., Ruiz-Vicente D., Areces F., Puente C., Carril J.C. (2017). ACTN3 genotype influences exercise-induced muscle damage during a marathon competition. Eur. J. Appl. Physiol..

[54] Peplonska B., Safranow K., Adamczyk J., Boguszewski D., Szymański K., Soltyszewski I., Barczak A., Siewierski M., Ploski R., Sozanski H. (2019). Association of serotoninergic pathway gene variants with elite athletic status in the Polish population. J. Sports Sci..

[55] Lutz B., Marsicano G., Maldonado R., Hillard C.J. (2015). The endocannabinoid system in guarding against fear, anxiety and stress. Nat. Rev. Neurosci..

[56] Slivicki R.A., Saberi S.A., Iyer V., Vemuri V.K., Makriyannis A., Hohmann A.G. (2018). Brain-permeant and-impermeant inhibitors of fatty acid amide hydrolase synergize with the opioid analgesic morphine to suppress chemotherapy-induced neuropathic nociception without enhancing effects of morphine on gastrointestinal transit. J. Pharmacol. Exp. Ther..

[57] Nwosu B.U., Kum-Nji P. (2018). Tobacco smoke exposure is an independent predictor of vitamin D deficiency in US children. PLoS ONE.

[58] Yuan L., Ni J. (2022). The association between tobacco smoke exposure and vitamin D levels among US general population, 2001–2014: Temporal variation and inequalities in population susceptibility. Environ. Sci. Pollut. Res..

[59] Küchler E.C., Tannure P.N., Falagan-Lotsch P., Lopes T.S., Granjeiro J.M., Amorim L.M.F. (2012). Buccal cells DNA extraction to obtain high quality human genomic DNA suitable for polymorphism genotyping by PCR-RFLP and Real-Time PCR. J. Appl. Oral Sci..

[60] Assis J., Pereira D., Gomes M., Marques D., Marques I., Nogueira A., Catarino R., Medeiros R. (2013). Influence of CYP3A4 genotypes in the outcome of serous ovarian cancer patients treated with first-line chemotherapy: Implication of a CYP3A4 activity profile. Int. J. Clin. Exp. Med..

